# Apolipoprotein profiling as a personalized approach to the diagnosis and treatment of dyslipidaemia

**DOI:** 10.1177/0004563219827620

**Published:** 2019-03-19

**Authors:** L Renee Ruhaak, Arnoud van der Laarse, Christa M Cobbaert

**Affiliations:** 1 Department of Clinical Chemistry and Laboratory Medicine, Leiden University Medical Center, Leiden, The Netherlands; 2 Department of Cardiology, Leiden University Medical Center, Leiden, The Netherlands

**Keywords:** Mass spectrometry, laboratory methods, cardiology, clinical studies, proteins, analytes

## Abstract

An elevated low-density lipoprotein cholesterol concentration is a classical risk factor for cardiovascular disease. This has led to pharmacotherapy in patients with atherosclerotic heart disease or high heart disease risk with statins to reduce serum low-density lipoprotein cholesterol. Even in patients in whom the target levels of low-density lipoprotein cholesterol are reached, there remains a significant residual cardiovascular risk; this is due, in part, to a focus on low-density lipoprotein cholesterol alone and neglect of other important aspects of lipoprotein metabolism. A more refined lipoprotein analysis will provide additional information on the accumulation of very low-density lipoproteins, intermediate density lipoproteins, chylomicrons, chylomicron-remnants and Lp(a) concentrations. Instead of measuring the cholesterol and triglyceride content of the lipoproteins, measurement of their apolipoproteins (apos) is more informative. Apos are either specific for a particular lipoprotein or for a group of lipoproteins. In particular measurement of apos in atherogenic particles is more biologically meaningful than the measurement of the cholesterol concentration contained in these particles. Applying apo profiling will not only improve characterization of the lipoprotein abnormality, but will also improve definition of therapeutic targets. Apo profiling aligns with the concept of precision medicine by which an individual patient is not treated as ‘average’ patient by the average (dose of) therapy. This concept of precision medicine fits the unmet clinical need for stratified cardiovascular medicine. The requirements for clinical application of proteomics, including apo profiling, can now be met using robust mass spectrometry technology which offers desirable analytical performance and standardization.

## Introduction

Individuals with risk factors for cardiovascular disease (CVD) (which include smoking, hypertension, diabetes mellitus (DM), central adiposity and dyslipidaemia) and patients suffering from CVD may be treated with a combination of life style modification and pharmacotherapy. Currently, cardiovascular risk stratification is, in part, based on classical lipid parameters cholesterol (C), triglycerides (TG), high-density lipoprotein-cholesterol (HDL-C) and low-density lipoprotein-cholesterol (LDL-C). Although guidelines outline an impressive list of preventive and therapeutic measures,^1^ risk stratification and treatment are not always successful in the long term. There are several indicators of sub-optimal patient management, including, but not limited to: (1) residual risk despite successful therapy, e.g. with statins,^2^ (2) underdiagnosis of CVD in women,^3^ and (3) the presence of atherogenic remnant disease that is hard to diagnose with current diagnostic procedures.^4^

**Figure 1.**
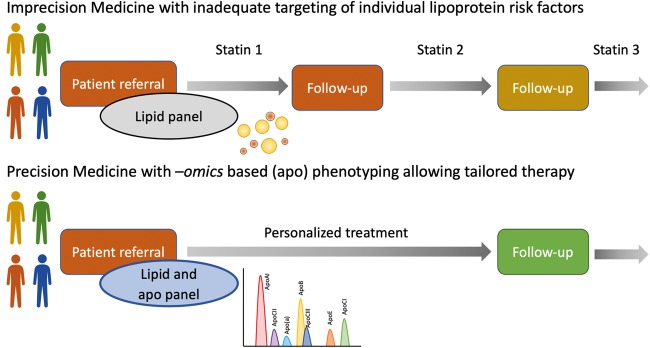
Conceptual view of the proposed paradigm shift from traditional, population-based medicine with prescription roulette due to statin intolerance/poor patient medicine concordance rather than lack of statin efficacy in attaining an LDL-C target and inadequate targeting of the individual lipoprotein risk factors, to precision medicine with personalized therapies, based on biology-driven test results.

Currently, the traditional evidence based medicine approach is used for risk stratification and therapy in patients with CVD. However, it is time for a paradigm shift from ‘reactive medicine’ that generally treats one particular item, e.g. LDL-C, to ‘proactive P4 medicine’ (predictive, preventive, personalized, and participatory), that is person-centric, based on disease mechanism and systems biology.^5,6^ In this holistic approach medical decisions are based on individual patient characteristics (including biomarkers) rather than on averages over a whole population. In this review, we summarize evidence for and propose the use of a multiplexed panel of apolipoproteins (apos) as an adjunct for cardiovascular risk stratification, to facilitate implementation of precision medicine (Figure 1).

A checklist to identify clinical management decisions linking biomarker testing to health outcomes was recently developed by the Test Evaluation Working Group of the European Federation of Clinical Chemistry and Laboratory Medicine (EFLM).^7^ The present review has been structured in line with this checklist and is organized into four domains: (a) identification of the unmet clinical need (deficiencies in current clinical care and described in the present section) (b) verification of the unmet clinical need i.e. are there existing biomarkers that might fill this unmet need (in particular the roles of apos in lipid metabolism as described in the section Biochemistry and pathobiology of apolipoproteins: Insight into dyslipidaemia and atherosclerosis), (c) validation of the intended use of the proposed biomarker marker (e.g. in sections on the current evidence for the role of apos in a clinical setting based on outcome studies at both the protein level and the genetic level) and (d) assessment of the feasibility of the new biomarker to influence clinical practice and health outcomes (recommendations for medical laboratories on the role of apo quantitation in the clinical care pathway, to be addressed in the section Embedding apolipoprotein profiling in cardiovascular precision clinical care pathways).^7^

### Residual CV risk beyond conventional CVD therapies

In the management of CVD the use of the most appropriate medication requires a clear definition of the underlying metabolic problem in a particular patient. Although the diagnosis may look simple, say diabetes mellitus type 2 (DM2) or ischemic heart disease, the most appropriate therapeutic measures may be difficult to select for each individual patient. Currently, clinical chemistry testing for dyslipidaemia in plasma/serum is, besides clinical history, echocardiography, electrocardiography and angiography, a cornerstone in the investigation of patients with CVD. The concentrations of cholesterol, TG, HDL-C and LDL-C are risk factors used to identify, stratify and characterize dyslipidaemia. This enables prescription of specific therapy directed towards these risk factors.

However, even if all risk factors present are treated, there remains residual CVD risk. In trials that studied statin vs. placebo therapy residual CVD risk was shown to be 25–40% in the statin-treated groups.^8–13^ Interestingly though, intensive statin therapy resulted only in a slight decrease in incidence of major CVD events.^14–16^ In 2005, Libby stipulated that the post-statin era suffers from a residual burden of cardiovascular mortality in about two-thirds of the patients with CAD, indicating the need for better understanding of factors contributing to this unexplained residual risk.^2^ Superko similarly concluded that a therapy that focusses on LDL-C reduction alone neglects other important aspects of lipoprotein metabolism.^17^ In the Multi-Ethnic Study of Atherosclerosis, the overall residual risk in statin-treated individuals was >17% in a 10 year period. Even in a subset of patients with a baseline LDL-C < 2.58 mmol/L, DM, LDL-particle size, hs-CRP and coronary artery calcification (CAC) were the most important predictors of residual risk.^18^ This raises the question of how to diminish residual risk.

### CVD in women

The treatment of CVD in women remains insufficient: compared to men, the prevalence and mortality of CVD in women is increasing. CVD generally manifests itself 10 years later in women than in men. Furthermore in addition to atherosclerotic coronary artery disease, non-atherosclerotic coronary artery disease may be an important cause of myocardial infarction, particularly in younger women. Risk factors in women include DM, endothelial dysfunction, leukocyte activation, platelet activation, renal dysfunction, and hypertension. The presenting symptoms are often non-specific in women, resulting in delayed diagnoses. In pre-menopausal women, severe manifestations of CVD, such as acute myocardial infarction (MI) and sudden death, are relatively rare. Women tend to develop asymptomatic heart failure secondary to CVD more frequently than men.^19^ Specific differences in CVD risk factors related to blood lipids have been described in women: (1) low HDL-C is more predictive for cardiovascular events in women than a high LDL-C; (2) high Lp(a) is a risk determinant for CVD in pre-menopausal and post-menopausal women <66 year; and (3) the concentration of total cholesterol seems to be associated with CVD only in pre-menopausal women and the concentration of TG only in older women.^20^

### Inadequate cholesterol-based tests do not identify atherogenic remnant disease

Remnant-cholesterol (remnant-C) includes primarily VLDL-C, IDL-C and Lp(a). Calculated remnant-C shows a skewed distribution with a tail towards higher concentrations in a general population.^21^ Hypertriglyceridemia (HTG) is associated with elevated concentrations of TG-rich lipoproteins (TRLs), apoB100, apoCIII and small dense LDL (sdLDL-C), in the setting of a normal or low concentration of LDL-C. Elevations of these (apo)lipoproteins, as are often observed in patients with DM or metabolic syndrome, are associated with increased CVD risk. In a model that included established risk factors, sdLDL-C was associated with incident CHD with a hazard ratio (HR) of 1.51 (95% confidence interval (CI), 1.21–1.88) for the highest vs. the lowest quartile. Even in individuals considered to be at low CVD risk based on their LDL-C concentrations, sdLDL-C predicted risk for incident CVD (HR 1.61; 95% CI, 1.04–2.49).^22^ LDL-C and remnant-C in ≈90,000 individuals from the Danish general population who were followed for up to 22 years were associated equally with risk of CHD and MI. Non-fasting remnant-C concentrations were associated stepwise with all-cause mortality ranging from HR of 1.0 (95% CI, 0.9–1.1) to HR of 1.6 (95% CI, 1.4–1.9) (P = 0.001), whereas LDL-C concentrations were associated with decreased all-cause mortality risk in a U-shaped pattern, with HRs ranging from 0.8 (95% CI, 0.7–0.8) to 0.9 (95% CI, 0.8–1.0) (P = 0.002). Only non-fasting remnant-C concentrations were associated with increased all-cause mortality risk.^21^

### Ineffective therapy due to average conclusions and average dosing not considering the individual patient

Statin group drugs to reduce LDL-C are widely used in the management of CVD. Clinical practice guidelines for the most part stipulate the titration of statin doses to achieve pre-specified ‘group’ (i.e. rather than individualized patient) LDL-C targets.^1^ A key question however is what is the personalized target for the patient? Treatment of comorbidities, such as other dyslipidaemias, DM, metabolic syndrome, hypertension or renal insufficiency, deserves attention in the individual patient. In other words, guidelines mention average target levels for the average patient, but an individual patient is likely not to be the average patient.

In recent years, several novel lipid lowering pharmacological therapies other than statins have been developed and results from clinical trials are now emerging.^23^ These include ezetimibe, a cholesterol absorption inhibitor, and the very potent PCSK9 inhibitors, that have already been licenced in Europe and the US.^24^ More recently, initial studies with anti-sense oligonucleotides (ASOs) blocking apoCIII and apo(a) have shown promising results.^25^ With these new therapies emerging, there is a need for specific guidance in selection of the most appropriate therapy and selection of the patients for this therapy.

We believe the time has come to change from generic therapy tailored towards the average patient to guidelines that address the specific goals to be met in the individual patient. Molecular markers which reveal the underlying biology and pathology can give better clues for definition and treatment of disease than average target levels of LDL-C. To that purpose, the use of a multiplexed apo panel is advocated over the traditional lipoprotein profile that is based on cholesterol and TG, and evidence for the use of such an apo panel is presented.

### Apolipoprotein quantitation by mass spectrometry

To accommodate the proposed paradigm shift to personalized healthcare, laboratory professionals explored alternative analytical strategies to define metabolic risk factors in individual patients. Mass spectrometry (MS) is a strategic (antibody-independent) technology for highly multiplexed protein quantification, that has recently entered the medical laboratory.^26,27^ Multiple clinically relevant (apolipo)proteins can simultaneously be enzymatically digested,^28^ upon which protein-specific signature peptides can be quantified using MS.

Newly developed (multiplex) biomarker methods should meet basic clinical chemistry principles.^29^ Criteria for imprecision and bias should be pre-defined, e.g., based on biological variation,^30^ and the methods should be robust and high-throughput to facilitate measurement of larger volumes of samples. The use of commutable and value-assigned serum-based calibrators should facilitate harmonization of assay methods and may contribute to a more rapid transition of biomarker discovery to clinical utility with benefit for the patient’s treatment and improvement of general health care.

We and others have now reported the development of liquid chromatography-mass spectrometry (LC-MS) based methods for the multiplexed quantitation of serum apos.^31–35^ We were able to show that our method demonstrates stable performance over a longer period of time.^36^ Moreover we showed that LC-MS based apoE phenotyping (qualitative analysis of apo ε2, ε3 and ε4 alleles) using unique signature peptides performs equally well compared to genotyping.^37^ These results show the strength of MS in metrology, as the technique allows for unequivocal identification and quantitation of well-defined measurands at the proteoform level through the use of specific and unique peptides. This has now also been recognized by the clinical chemistry community, as an IFCC working group has been initiated to establish metrological traceability and standardization of apo quantitation and phenotyping using MS (http://www.ifcc.org/ifcc-scientific-division/sd-working-groups/wg-apo-ms/).

### Inadequate standardization of Lp(a) measurement

Apo(a) is a heterogeneous glycoprotein with large size heterogeneity ranging from 200 to 800 kDa, due to a size polymorphism. The measurement of Lp(a) is indicated in intermediate CVD risk patients, patients with inherited dyslipidaemias and patients with premature vascular disease.^1,38^ However, the size polymorphism of apo(a) hampers the accurate quantitation op Lp(a) in g/L,^39,40^ as well as standardization. While Lp(a) has been known to be a risk factor for CVD for a long time, the poor quality of quantitative tests has hampered adoption of Lp(a) in clinical care pathways so far. The National Heart Blood and Lung Institute Working Group on Future Research Directions on Lipoprotein(a) and Cardiovascular Disease therefore recently recommended the development of a standardized measurement of Lp(a), in which apo(a) values should be quantified in nmol/L.^41^ Here too, MS seems to be an excellent technology to achieve this, as selection of a non-kringle IV-2 peptide would allow for quantitation of apo(a) at the molar level.

## Biochemistry and pathobiology of apolipoproteins: Insight into dyslipidaemia and atherosclerosis

### ApoB100

Human apoB100 is a 4536-amino acid secretory glycoprotein, and a single molecule is present in each VLDL particle when secreted by the liver. Lipoprotein lipase (LPL) and hepatic lipase (HL) are responsible for lipolysis of TG of the VLDL particle in the circulation, leading to formation of VLDL-remnants. Although VLDL-remnants (also called IDL) are predominantly cleared by the liver (through apoE-mediated pathways, see below) a large part of VLDL-remnants is converted – via IDL – into LDL by further lipolysis of TG. ApoB100 is responsible for uptake of VLDL, IDL and LDL by the hepatic LDL-receptor (LDLR).^42^ Generally, the concentration of LDL in the circulation is relatively high, compared to that of VLDL and IDL. LDL is relatively poor in TG and rich in cholesterol, whereas VLDL and IDL are particles that are relatively rich in TG.

Each particle of VLDL, IDL and LDL contains – in theory – one copy of apoB100. In the small intestine, a truncated form of apoB is formed corresponding to its N-terminal 48% (apoB48), which directs the formation of CMs. These TG-rich and cholesterol-poor particles undergo lipolysis of TG in the circulation, leading to uptake of CM-remnants by the liver and – in certain circumstances – accumulation of CM-remnants in the blood.^43^

A high level of total cholesterol and particularly of LDL-C is a strong risk factor for atherosclerosis underlying several diseases such as CHD. In the initiation of atherosclerosis crucial events are the subendothelial retention and modification of apoB100-containing lipoproteins. However, not only abundancy of LDL but also abundancy of remnant particles trigger the atherosclerotic process, although the latter by mechanisms that are currently understood poorly.^44^ LDL-lowering drugs (e.g., statins, ezetimibe and PCSK9 inhibitors) are currently the most effective lipid lowering therapies in routine clinical use and are shown to reduce coronary events and improve survival, which is attributed to the reduction in LDL-C.

**Figure 2.**
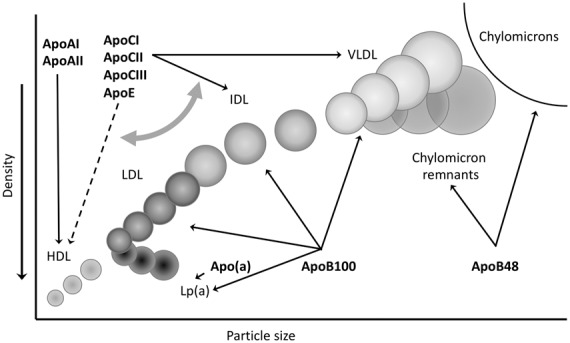
Density and particle size of the different lipoproteins. Their major apolipoprotein constituents are indicated.

Apart from VLDL, IDL and LDL, a fourth lipoprotein type, Lp(a), carries apoB100 (Figure 2). Lp(a) is composed of an apoB100-containing LDL-like particle, covalently linked to the plasminogen-like glycoprotein apo(a). Lp(a) is a highly prevalent genetic risk factor for CVD and calcific aortic valve disease.^45^

In patients with high concentrations of sdLDL, the risk of CHD is best assessed by apoB concentrations (thus including apoB100 and apoB48), as the presence of sdLDL is not reflected by elevated LDL-C concentrations. Thus, an apoB measurement includes, besides LDL, also Lp(a), IDL, VLDL and CM-remnants,^46^ and is recommended as (1) assessment of risk of CHD and (2) target of lipid-lowering therapy.^47,48^

### ApoCI

ApoCI is a single-chain polypeptide of 57 amino acids. ApoCI is produced in the liver and is a constituent of VLDL and HDL. ApoCI is an inhibitor of LPL activity, and inhibits the binding of VLDL to LDL-receptor related protein (LRP),^49,50^ as well as the apoE-mediated binding of VLDL to the LDLR and the LRP.^51,52^ ApoCI is a potent activator of lecithin:cholesterol acyltransferase (LCAT),^53,54^ leading to elevated HDL-cholesterol ester concentrations.^55–57^ In apoCI-deficient mice, the primary metabolic defect is an impaired VLDL uptake by the liver in vivo.^58^ Interestingly, whereas overexpression of human *APOC1* in transgenic mice predominantly inhibits the uptake of VLDL by the liver, the absence of endogenous apoCI in mice appears to have the same effect, although to a lesser extent. ApoCI has been reported to inhibit cholesteryl ester transfer protein (CETP) activity.

### ApoCII

ApoCII is a single chain polypeptide of 79 amino acids. ApoCII is an essential cofactor of LPL, the rate-limiting enzyme for the hydrolysis and removal of TG in CMs and VLDL.^59^ Like apoCI, but less effectively, apoCII inhibits the apoE-mediated binding of VLDL to the LRP.^49^ The plasma TG concentration is independently associated with the apoCII concentration in a model including waist, circumference, apoCIII, apoE, glucose, insulin and mean LDL size in hyperlipidaemic patients.^60^

Statin treatment resulted in significant apoCII reduction by ≈20%.^61–63^ It was found that the apoCII-lowering effect of rosuvastatin occurred only in the subgroup with TG concentration ≥1200 mg/L.^63^

In a study of 27 patients with primary HTG (TG >3500 mg/L) who received atorvastatin (20 mg/day or 80 mg/day) for four weeks, dose-dependent reductions of total cholesterol and TG were observed. In addition significant reductions in apoCII (by 28% and 42%), apoCIII (by 18% and 30%), and apoE (by 37% and 49%) were observed.^62^

Individuals who are completely deficient in apoCII have chylomicronaemia and grossly elevated plasma TG concentrations, although their parents, who have half normal plasma concentrations of apoCII, have normal TG concentrations.^64^ However, transgenic mice overexpressing human *APOC2* had HTG, due to an accumulation of TG-rich VLDL in their circulation, caused by an impaired clearance of VLDL-TG, consistent with a defective LPL-mediated hydrolysis of VLDL-TG.^65^ These results are in contrast with the LPL-activating action of apoCII: it may be that apoCII is an activator of LPL at low apoCII concentration, whereas a high apoCII concentration directly inhibits VLDL lipolysis.^49^ Thus, both an excess and a deficiency of apoCII are associated with reduced LPL activity and HTG.^66^

### ApoCIII

ApoCIII is a 79-amino acid polypeptide that is produced mainly in the liver and to a lesser extent in the intestines. ApoCIII is a significant component of TRLs and a minor component of HDL.^67^ ApoCIII exerts its impact on TG metabolism by four distinct mechanisms: (1) apoCIII functions as an inhibitor of HL and LPL, key enzymes that catalyse the hydrolysis of TG in VLDL and CMs;^68–70^ (2) apoCIII acts to retard apoE-mediated hepatic uptake of TRLs;^71,72^ (3) apoCIII serves to facilitate VLDL-TG assembly and secretion from the liver;^73–76^ and (4) apoCIII completely abolishes the apoB100-mediated binding of lipoproteins to the LDLR, probably due to a masking of the receptor domain of apoB100 by apoCIII.^77,78^

In the fasting state, apoCs are mainly associated with HDL, whereas in the fed state, as well as in patients with HTG, the apoCs preferentially redistribute to the surface of CMs and VLDL particles.^79^ Elevated plasma apoCIII concentrations are associated with augmented production and retarded clearance of TRLs, characteristic of HTG. Plasma apoCIII concentrations were positively correlated with (1) plasma TG and (2) VLDL-TG.^80^

Fibrates, a class of drugs that reduce plasma TG concentrations, effectively decrease apoCIII synthesis rate in humans^81^ as well as APOC3 mRNA concentrations in isolated human hepatocytes and rat livers via a peroxisome proliferator activated receptor (PPAR)-dependent pathway.^82–84^ The development of ASOs targeted to the hepatic mRNA of apoCIII holds considerable promise, as dose-dependent reductions in TG levels of up to 80% are attainable.^85^

### ApoE

ApoE, a 299-amino acid polypeptide, is a ligand for the LDLR and LRP, binds to (hepatic and endothelial) cell surface heparin sulphate proteoglycans, and plays a key role in the receptor-mediated uptake of apoB100-containing lipoproteins including remnant lipoproteins.^79^ In addition to its well-established function, apoE is proposed to modulate TG lipolysis.^86^ ApoE can specifically inhibit the LPL-mediated hydrolysis of emulsion TG both in vitro and in vivo and inhibits the LPL-mediated processing of TRLs, including VLDL-TG.^87–89^ Plasma apoE is synthesized primarily by liver hepatocytes, which account for ≈75% of the body’s apoE production. Various cell types throughout the body, including macrophages, also synthesize apoE.^90^ The hepatic uptake of VLDL-remnants and CM-remnants is facilitated by apoE, but is inhibited by apoCI, apoCII and apoCIII.^91^

Epidemiologic studies have shown that apoE polymorphism is associated with interindividual variations in plasma lipid concentrations as well as susceptibility to atherosclerosis.^86,91^ ApoE4 is associated with higher plasma total cholesterol, LDL-C, and apoB100 concentrations than apoE3. In addition, apoE4 is proposed to be an independent risk factor for CHD regardless of its effect on plasma cholesterol concentrations.^92–94^ On the other hand, homozygotes of apoE2, insofar as they do not develop type III hyperlipoproteinaemia (also called dysbetalipoproteinaemia), show lower plasma total cholesterol and LDL-C concentrations, lower apoB100 concentrations, and higher plasma TG concentrations than homozygotes of apoE3. Homozygotes of apoE2 demonstrate higher postprandial plasma TG concentrations than those of apoE3 and apoE4.^95,96^ Furthermore, apoE2 is proposed to reduce the risk of CVD.^97^ However, homozygotes for apoE2 who develop type III hyperlipoproteinaemia due to secondary precipitating factors are susceptible to CVD.^98,99^

### Apo(a)

Lp(a) is composed of an apoB100-containing LDL-like particle, covalently linked to the plasminogen-like glycoprotein apo(a). Lp(a) is highly polymorphic in size due to the number of kringle IV type 2 (KIV2)-encoding sequences, giving origin to >40 apo(a) isoforms varying in number among individuals and populations. Although its exact role in lipid metabolism is unknown, Lp(a) is a highly prevalent genetic risk factor for CVD and calcific aortic valve disease (CAVD). Lp(a) concentrations in the atherothrombotic range are generally accepted as >300 to 500 mg/L (>75 to 125 nmol/L). Such concentrations affect 20% to 30% of the global population, with possibly higher incidence in patients with established CVD and CAVD.^41^

The atherogenicity of Lp(a) is caused by interference with the fibrinolytic system, the affinity to secretory phospholipase A2, the interaction with extracellular matrix glycoproteins and the binding to scavenger receptors on macrophages. An association between high Lp(a) and incidence of venous thromboembolism (VTE) has been described. Apo(a) KIV-2 repeat number was significantly lower in VTE patients (excl. hereditary and acquired thrombophilia) than in controls (11 vs. 15, respectively). KIV-2 repeat number was independently associated with VTE.^100^ Elevated Lp(a) concentration has a strong association with angiographically documented CAD^45,101^ and with risk of cardiovascular death, non-fatal MI and ischemic stroke.^102^ Nordestgaard et al. advocate screening for elevated Lp(a) in those with intermediate or high CVD/CHD risk, and use of pharmacotherapy for Lp(a) and CVD/CHD risk reduction, accepting a desirable concentration <500 mg/L.^103^

### Apo A-I

ApoA-I is the major apo in HDL, accounting for 70% of all HDL-associated proteins, and mediates many of the anti-atherogenic functions of HDL. ApoA-I is produced both in the liver and intestines, and is responsible for initiating reverse cholesterol transport, whereby excess cholesterol in peripheral tissues is carried back to the liver for excretion. Patients with low concentrations of apoA-I (<1.2 g/L) are more likely to have CVD than those with high apoA-I concentrations (≥1.6 g/L).^104^

As with LDL, the cholesterol content of HDL particles varies among patient types and is influenced by plasma TG concentrations; patients with high TG tend to have low apoA-I concentrations. Nonetheless, it is unclear whether apoA-I alone is a predictor of CVD risk independently of its association with HDL.^105^

## Genetic evidence for the role of apolipoproteins in CVD

Three types of studies are typically used to determine whether a certain parameter is a risk factor for CHD. These studies include primary evidence through observational studies, randomized controlled trials (RCT), and lately Mendelian randomization trials (MRTs).^106^ Because genetic evidence from MRTs is most compelling, in this review we specifically focus on evidence for the role of apos in CHD obtained from such study designs. Mendelian randomization was first proposed in 1986 to evaluate whether the association between cholesterol concentrations and cancer was really causative using apoE genotypes (ε2, ε3, ε4),^107^ and has been reviewed extensively.^108^ It is a method in which measured variation in genes of interest is used to evaluate the effects of a measure of exposure (e.g., concentrations of cholesterol or TG) on disease states (e.g., incidence of CHD).^109^ The design is highly resistant to reverse causation and confounding, which often impede or mislead epidemiological studies. Most striking are occasions where observational epidemiological studies have highlighted a seemingly substantial causal association that later failed to be confirmed in large-scale RCTs. An example is the hypothesis that β-carotene protects against cancer, which was derived from several observational epidemiologic studies.^110^ This hypothesis was tested in the ATBC and CARET studies, both RCTs in which a 16%^111^ and 28%^112^ increased incidence of lung cancer was reported in individuals treated with β-carotene, respectively. Genotypes are randomly inherited from parents to offspring, known as Mendelian randomization, and as such, the genotype distribution used in RCTs should be unrelated to confounders that may affect conventional observational studies.^108^ Moreover, a genetic polymorphism can, by definition, only be causative to a change in the measure of exposure, thus avoiding reverse causation. However, genetic pleiotropy may confound results from MRTs, and a number of methods to identify pleiotropy in MRTs have been described.^113^

### ApoB

There is a large body of evidence for a direct implication of LDL and other apoB-containing particles (VLDL, IDL and Lp(a)) on the development of CVD in a concentration dependent manner.^114^ Specifically, a number of meta-analyses, such as the Prospective Studies Collaboration and the Emerging Risk Factors Collaboration, have provided evidence that there is an association between exposure to LDL and risk of CVD.^115,116^ Even though mutations in the APOB gene might be prone to pleiotropy due to association with other lipoproteins or risk factors for CVD, some MRT studies have used single nucleotide polymorphisms (SNPs) in APOB to evaluate CVD risk. Kathiresan et al. evaluated the role of LDL-C and HDL-C in CVD through common SNPs in nine genes.^117^ APOB SNP rs693 was shown to be individually associated with LDL-C concentrations in 5287 subjects of the cardiovascular cohort of the Malmo¨ Diet and Cancer Study. Subsequently, a panel of nine SNPs from nine genes, including APOB rs693 could be associated with incident CVD, providing evidence for the causal association of LDL-C concentrations with CVD. In a similar approach, APOB rs562338 was included in a multi-SNP panel to assess the relation between LDL-C and CVD risk.^118^

Besides the MRTs, there is evidence for the role of apoB from observational studies towards inherited forms of CVD. Individuals with a loss-of-function mutation in the apoB gene typically have familial hypercholesterolemia (FH), which is characterized by substantially elevated concentrations of LDL-C and early-onset CVD, specifically CAD (e.g. Pang et al.^119^), further validating the causative role for apoB in CVD.

### ApoCII

The second most frequently reported cause of chylomicronaemia, often resulting in severe pancreatitis, is the presence of homogeneous APOC2 loss-of-function mutations.^120^ Indeed, several case reports indicate chylomicronaemia and decreased concentrations of apoCII in individuals with APOC2 loss-of-function mutations.^121–125^ However, so far, these numbers are too small for full observational studies, and MRTs have not yet been conducted. In 38 patients with very premature STEMI, 4 patients with low apoCII concentrations (≤5.0 mg/L) had worse reinfarction-free or revascularization-free survival than those with apoCII >5.0 mg/L during follow-up of 10 years (Figure 5(a) and (b)).^126^ These findings point towards a role for apoCII in CVD, and might warrant further evaluation of the role of apoCII in an MRT.

### ApoCIII

There is a long-lasting debate on whether or not TG concentrations are a risk factor for CHD, and several studies indicated that TG concentrations are associated with CHD. However, adjusting for confounding variables (e.g., DM) weakened these associations. Because apoCIII is one of the major constituents of TRLs, MRT studies were performed to further evaluate the relationship between TG concentrations and CHD risk.

In one study, four rare apoCIII variants were identified to be associated with lower TG concentrations: nonsense mutation R19X, two splice-site mutations (IVS2 + 1G→A and IVS3 + 1G→T) and a missense mutation A43T. Carriers of these mutations had 39% decreased TG concentrations and circulating concentrations of apoCIII were 46% lower. When these mutations were subsequently tested in 110,097 individuals from several studies, carriers had a 40% reduction in CHD.^127^ In a similar study, three apoCIII variants could be associated with a 44% reduction in TG concentrations. In a Danish cohort of 75,725 individuals, it was subsequently observed that carriers of these variants had a 41% reduction in CHD.^128^ These studies clearly indicate that reduction of apoCIII reduces CHD risk. However, whether this reduction in CHD is TG mediated is unsure, as reduced concentrations of apoCIII have also been associated with lower concentrations of LDL-C.^129^

Further evidence for the role of apoCIII as a risk factor for CHD can be found in a study which found that about 5% of the Lancaster Amish are heterozygous carriers of an APOC3 null mutation (R19X). Lower TG and LDL-C concentrations were observed in mutation carriers and the incidence of subclinical atherosclerosis was decreased, indicating a cardioprotective effect of apoCIII deficiency.^130^ The same mutation has subsequently been found to be enriched in an isolated Greek population, in which it could be associated with reduced TG concentrations.^131,132^

### Apo(a)

Apo(a) is the characteristic polymorphic protein carried by Lp(a) particles, and evidence for the role of Lp(a) as a risk factor for CVD has recently been reviewed.^45^ A number of observational studies have clearly identified Lp(a) concentration to be associated with CVD incidence,^102,133,134^ indicating a potential causal role for Lp(a). In a meta-analysis in which 36 studies with 7385 cases and 8514 controls were included, small apo(a) isoforms (approximately 22 or fewer KIV2 repeats) were shown to be associated with a two-fold increased risk for CVD when compared to larger apo(a) isoforms.^135^

To further assess the role of Lp(a) particles in CVD risk, MRTs have been performed (e.g. ^133,136–138^), and results were recently reviewed.^45^ Here the most important, and novel findings are summarized. In 2009, LPA SNPs rs10455872 and rs3798220 were shown to be associated with Lp(a) concentration. These same SNPs were subsequently shown to also be associated with CVD risk,^136^ thus directly indicating a causal effect for apo(a) concentration on CVD. These results were corroborated by Kamstrup et al.^133^ Recently, the Mendelian randomization strategy was also used to estimate the required change in plasma Lp(a) concentration that should therapeutically be reached to produce a clinically meaningful reduction in CVD risk.^138^ A panel of 43 genetic variants, which explained 51–63% of the variance in Lp(a) concentration, was used to predict Lp(a) concentration, and it was modelled that each 100 mg/L lower Lp(a) was associated with a 5.8% lower CVD risk. By comparing the risk reduction with the same parameter for LDL-C risk reduction, it could be inferred that an 1 g/L reduction in apo(a) concentrations would provide a similar reduction in CVD risk as a 1 mmol/L reduction in LDL-C concentrations, which is generally regarded clinically meaningful.^138^

There is an inverse relation between apo(a) concentration and the number of apo(a) KIV2 repeats. However, there is large interindividual variation in this correlation,^45^ which is indicated by a study that showed that the size variation of apo(a) could explain 17% of the apo(a) concentration in Sudanese, but 77% in Mexicans.^139^ Because of this correlation, it has long been unclear whether just Lp(a) concentration, or also apo(a) KIV2 number influences CVD risk. To address this question, Salaheen et al. performed an MRT by identifying genetic variants associated independently with either apo(a) KIV2 number (rs2457564) or apo(a) concentration (rs3777392) in the Pakistan Risk of Myocardial Infarction Study (PROMIS).^140^ Genetic data from 60,801 CHD cases and 123,504 controls from the CARDIoGRAMplusC4D consortium revealed that the OR for MI was 0.96 per 1SD increment of apo(a) KIV2 number, while an OR of 1.27 was observed per 1SD increment of apo(a) concentration. Because the SNPs were chosen to be specific for their apo(a) characteristic, it could be concluded that both apo(a) KIV2 numbers as well as apo(a) concentration are independent causal risk factors for CVD.^140^

### ApoE

**Figure 3.**
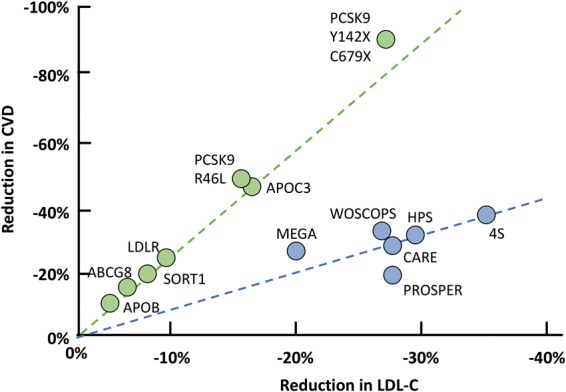
Overview of the relation between reduction in LDL-C and reduction in CVD in genetics and intervention studies. A lifetime reduction of LDL-C by genetic traits (studies indicated by the green dots, mutations indicated) reduces the risk of CVD two to three times more than pharmacological reduction of LDL-C by statin therapy during an average of five years (studies indicated by the blue dots).*Source:* reproduced with permission from Elsevier.^129^

In a study evaluating the effect of long-term exposure to lower LDL-C concentrations on the risk of CVD, nine SNPs in six genes, among which APOE rs4420638, were studied.^141^ The APOE SNP was shown to be associated with reduced LDL-C concentrations in a meta-analysis involving 126,788 samples. In a subsequent meta-analysis, these nine SNPs, including APOE rs4420638 were associated with a decreased risk of CHD. The correlation of proportional CHD risk reduction and lower LDL-C revealed 18% CHD risk reduction per 0.25 mmol/L lower LDL-C (≈72% lower risk per mmol/L lower LDL-C).^141^ In a meta-analysis of 26 statin trials, treatment with a statin was associated with a 24% reduction of CHD risk per mmol/L reduction of LDL-C. Thus, compared with treatment with a statin **later in life**, prolonged exposure to lower LDL-C beginning **early in life** was associated with a two- to three-fold greater reduction in the risk of CHD for each unit lower LDL-C (Figure 3).^129,141^ This was further corroborated by Kathiresan et al., who monitored the same APOE rs4420638 SNP to study the relation between LDL-C and CVD risk in the Malmo¨ Diet and Cancer Study.^117^

ApoE has three common alleles encoded by the *APOE* gene. These alleles occur at different frequencies in humans (ε2, 5–10%; ε3, 65–70%; and ε4, 15–20%) and give rise to three homozygous (apoE2/2, apoE3/3, and apoE4/4) and three heterozygous (apoE3/2, apoE4/2, and apoE4/3) phenotypes.^142^ The structural basis for the three isoforms occurs through amino acid interchanges (single base changes in the apoE gene) at residues 112 and 158: apoE2 has cysteine at both sites, apoE4 has arginine at both sites, and apoE3 has cysteine (Cys)-112 and arginine (Arg)-158.^143^ The hypothesis that the APO ε2/3/4 genotype affects plasma Lp(a) concentration was tested by Kritharides et al.^144^ They found that the APOE ε2 genotype is a strong determinant of low Lp(a) concentrations, but the APOE ε2 genotype does not modify the causal association of Lp(a) with MI or CAVD.^144^

### ApoA1

Haase et al. sequenced the regulatory and coding regions of APOA1 in 190 individuals from the Copenhagen City Heart Study with the lowest 1% (n = 95) and highest 1% (n = 95) apoA-I concentrations.^145^ Genotype combinations of two SNPs were associated with increases in apoA-I and HDL-C concentrations of up to 6.6 and 8.5%, respectively. Although these genotypes would theoretically predict reductions in 9 and 8% for CHD risk and 14 and 12% for MI risk, these genetic variations of APOA1 did not associate with decreased risk of CHD or MI in daily practice. The authors suggest that the atheroprotective effects of high concentrations of apoA-1 and HDL-C in epidemiological studies may not be due to the apoA-I and HDL-C elevations itself, but may be caused by the concomitant reduction in TG concentrations and thus by the concomitant reduction in atherogenic remnant lipoproteins.^145^

### ApoA-IV

ApoA-IV has been observed to be associated with lipid concentrations, renal function, and adiposity- and diabetes-related parameters. Mack et al. conducted bidirectional Mendelian randomization analysis to assess the causal relationship of apoA-IV with these phenotypes.^146^ These causal relationships were found with estimated glomerular filtration rate, and serum TG concentration, independently from HDL-C and LDL-C concentrations. Evaluating the inverse direction of causality revealed a possible causal association of apoA-IV on HDL-C.^46^

### ApoA-V

The Triglyceride Coronary Disease Genetics Consortium and Emerging Risk Factor Collaboration assessed the −1131T>C (rs662799) promoter polymorphism of the apoA-V (APOA5) gene in relation to TG concentration, several other risk factors, and risk of CHD.^147^ −1131T>C was modestly associated with lower HDL-C, lower apoA-I, and higher apoB, and strongly associated with elevated TG. The OR for CHD was 1.18 (95% CI 1.11–1.26; p = 2.6 × 10^−7^) per C allele. −1131T>C was significantly associated with higher VLDL particle number and smaller HDL particle size, factors believed to mediate the effects of TG.^147^

In an evaluation of the contributions of rare mutations to MI risk in the population, Do et al. sequenced the protein-coded regions of 9793 genomes from patients with MI at an early age (≤50 years in males and ≤60 years in females) along with MI-free controls.^148^ Carriers of rare APOA5 mutations (1.4 of cases vs. 0.6% of controls) were at 2.2-fold increased risk of MI. APOA5 mutation carriers had higher plasma TG, which suggested that elevated concentrations of TRLs contribute to MI risk.^148^

## Apolipoprotein profiles and cardiovascular risk: Evidence from outcome based studies

**Figure 4.**
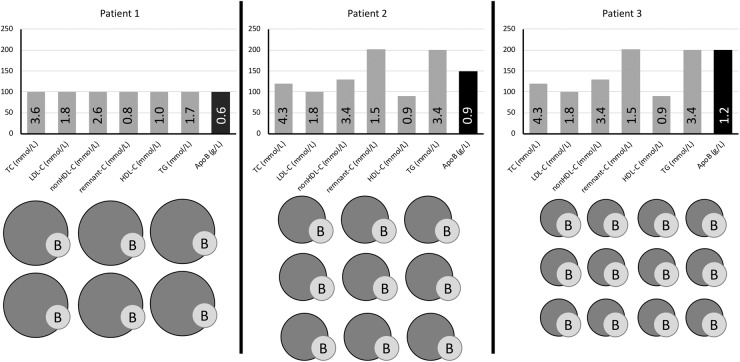
Relative LDL particle size and plasma lipids/lipoprotein concentrations in three patients with identical low LDL-C (1.8 mmol/L) but with discordant non-HDL-C and apoB with regard to desirable treatment targets for very high-risk patients. Patient 1 has all three targets at goal and normal numbers of (predominantly larger sized) LDL particles. Patient 2 with moderate HTG has discordant non-HDL-C above target (2.6 mmol/L) because of increased remnant-cholesterol. Patient 3 also has moderate HTG and increased remnant-cholesterol but concurrently higher apoB concentration than patient 2 because of a high number of sdLDL particles not detected by standard LDL-C measurement.*Source:* reproduced with permission from American Association for Clinical Chemistry.^46^

Besides evidence for the role of apos in CVD from genetics studies, there is now also ample literature describing associations between protein concentrations and CVD outcome. Initially, these studies were performed using immunoassays, but recently studies using MS have emerged.^149^ Specifically, several studies have attempted to improve current LDL-C measurements by targeting apoB100, the major apo of LDL. In a large cohort study of 175,553 individuals Walldius et al. reported that the relative risk (RR) of MI per 1SD increment of LDL-C was substantially reduced after additional adjustment for apoB100; in contrast, apoB100 was still associated with substantial risk when adjusted for LDL-C, indicating that the risk of atherosclerotic disease is best predicted by apoB100, instead of LDL-C and non-HDL-C.^150^ Pischon et al. found that apoB was more strongly related to CHD risk than was non-HDL-C. This is in line with previous studies showing that apoB100 is superior to non-HDL-C in predicting subclinical atherosclerosis. Based on the now disputed assumption that each LDL particle contains one apoB100 molecule, these findings led Pischon et al. to state that ‘*direct measurement of the concentration of atherogenic particles is more biologically meaningful than the measurement of the cholesterol concentration contained in these particles*’.^151^ In a meta-analysis based on 12 published epidemiological studies that contained estimates of the relative risks of non-HDL-C and apoB for CVD, it was concluded that over a 10-year period, a non-HDL-C strategy would prevent 300,000 more events than an LDL-C strategy, whereas an apoB100 strategy would prevent 500,000 more events than a non-HDL-C strategy.^152^ These findings indicate that apoB100 is superior to LDL-C and non-HDL-C as a predictor of CVD risk. These results are consistent with CHD risk being more closely related to the number of atherogenic apoB100 particles than to the mass of cholesterol within them, as previously stated (Figure 4).^47^

**Figure 5.**
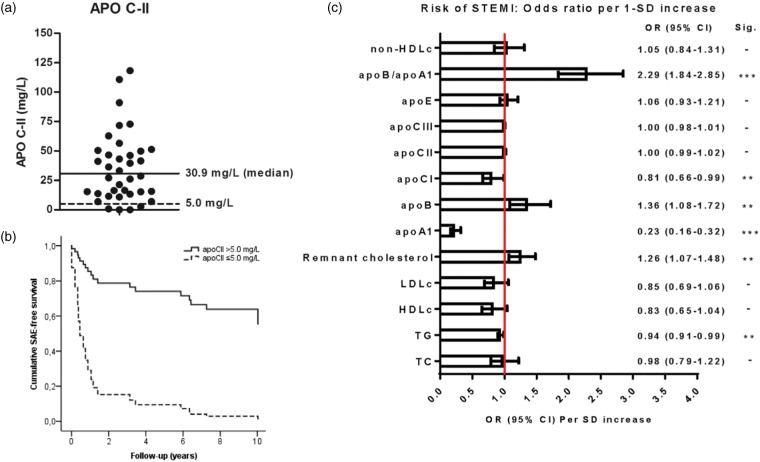
(a) ApoCII concentration in 38 patients with very premature STEMI. (b) In this group of patients with very premature CAD, 4 patients with apoCII ≤5 mg/L had a high risk of reinfarction or revascularization over ten years compared to those with apoCII >5 mg/L. (c) In the MISSION Intervention Study the serum concentrations of apoA1, apoB and the apoB/apoA1 ratio were strongly associated with risk of STEMI.*Source:* reproduced with permission from Elsevier^126^ and SpringerNature.^156^

The risk of CVD is inversely proportional to HDL-C and apoA-I concentrations, but this seems to be mostly relative to LDL-C.^153^ Therefore, quantitation of apoA-I may be most useful in conjunction with apoB100 to assess the balance between atherogenic and atheroprotective cholesterol transport. A higher apoB100/apoA-I ratio likely indicates that more atherogenic particles are circulating in plasma leading to more plaque build-up in arteries, atherosclerosis and higher risk of CVD events.^154^ Compared to other lipid ratios, the apoB100/apoA-1 ratio may be more accurate in risk prediction, particularly among high-risk individuals, than high LDL-C and low HDL-C concentrations.^155^ Concordantly, we reported recently that the apoB/apoA-1 ratio was strongly associated with risk of ST-segment elevation myocardial infarction (STEMI) (Figure 5(c)).^156^

While apoB100 is mostly associated with LDL particles, and apoA-I partially reflects HDL-C, the situation for apos associated with TRLs is more complicated. For instance, in normolipidaemic subjects, apoCII is mainly distributed in HDL, whereas in subjects with HTG, apoCII is predominantly found in the VLDL and LDL. TG concentrations are generally inversely related to HDL-C, and for a long time, it was assumed that the measurement of HDL-C enabled calculation of CVD risk. However, there is now ample evidence from genetic studies (Genetic evidence for the role of apolipoproteins in CVD Section) that not low HDL-C, but high concentrations of TRLs are a causal factor for CVD.^106^ Therefore, interest in TRLs and TRL-associated apos, such as CI, CII, CIII and E,^49^ as markers for CVD risk has surged.

In 2002, Gerber et al. reported that increased apoCII concentrations are associated with CHD after adjustment for cardiovascular risk factors in a case–control study including 352 CHD patients and 395 controls.^157^ This evidence supports an association between apoCII and CVD risk, but causality between apoCII concentrations and CVD was not established.^66^ Interestingly, we recently identified a phenotype of relatively young women with a combination of very low apoCII (≤5 mg/L), normal TG concentrations, and low concentrations of LDL-C, apoB100, and apoE who presented with STEMI. Despite their low a priori risk for CAD, these women presented with MI and had a high relative risk of 10-year reinfarction or revascularization.^126^ These finding further point towards a role for apoCII in CVD.

In a nested case–control study in the prospective EPIC-Norfolk study comprising 2711 apparently healthy study participants, of whom 832 subsequently developed CAD, Van Capelleveen et al. demonstrated that increased apoCIII concentrations were significantly associated with incidence of CAD.^158^ The significance was retained after adjustment for traditional CAD risk factors, but it was proposed that elevated concentrations of remnant lipoproteins, sdLDL and low-grade inflammation could explain the association with CAD.^158^

Recently, Pechlaner et al.^149^ used a targeted MS-based method to evaluate the association between apoA-I, apoA-II, apoA-IV, apoB100, apoCI, apoCII, apoCIII, apoD, apoE, apoH, apoL-I, apoM and apoJ with incident CVD in 688 participants of the Bruneck Study. During 10 years follow-up, the apos that were most significantly associated with incident CVD were apoCII (hazard ratio (HR) per 1 SD: 1.40; 95% CI, 1.17–1.67), apoCIII (HR per 1 SD: 1.38; 95% CI, 1.17–1.63), and apoE (HR per 1 SD: 1.31; 95% CI, 1.13–1.52). These associations remained significant even after adjustment for traditional risk factors, and demonstrate the advantage of using apos to identify dyslipidaemic states, particularly related to TRLs.^149^

Further evidence for the direct role of apoCIII in the development of CVD comes from the novel anti-APOCIII ASO therapy with volanesorsen. ApoCIII inhibition by volanesorsen reduced plasma concentrations of apoCII, apoCIII, and TG, without affecting concentrations of apoB100.^149,159,160^ Whether targeting of apoCIII in patients with elevated concentrations of TRLs will reduce risk of CVD is subject of ongoing studies, but a recent report on the use of volanesorsen in individuals with familial chylomicronaemia syndrome, a rare genetic disorder characterized by marked chylomicronaemia leading to pancreatitis, indicated a significant reduction in disease burden.^161^

There is very limited evidence for a direct causal role of apo(a) in CVD, mostly because direct measurement of apo(a) is not performed routinely. However, a 1:1 stoichiometry of apo(a) on Lp(a) particles is assumed, and a large body of evidence exists for the association between Lp(a) concentrations and the risk for CAD, regardless of other risk factors. For example Kostner et al.^162^ have estimated that CAD risk is 2.3 times higher in patients with Lp(a) concentrations >500 mg/L, while Riches and Porter have calculated that risk was twice greater for Lp(a) concentrations >200 mg/L.^163^ In a meta-analysis of 40 prospective studies with 58,000 participants, a two-fold increase in the risk for developing CAD and cerebral vascular accident was found in individuals with smaller apo(a) isoforms, regardless of the Lp(a) concentration and the classical risk factors.^135^

Recently, an anti-APOA ASO (IONIS-APO(a)-LRx) therapy has been developed. On treatment, Lp(a) concentrations fell by an average of 68%.^164^ Studies further evaluating the efficacy for this therapy in lowering CVD incidence are currently ongoing. Chronic lipoprotein apheresis is an alternative treatment that was previously developed, and 154 subjects with isolated Lp(a)-hyperlipoproteinaemia with progressive CVD who underwent apheresis showed reduced Lp(a) concentrations by 68%, associated with a decline of major adverse cardiac events by 78% after five years.^165^

## Embedding apolipoprotein profiling in cardiovascular precision clinical care pathways

### Conventional clinical care pathway

Currently, the standard procedure for patients that present with symptoms of CVD is to monitor the patient’s lipid profile by determining the concentrations of total cholesterol, TG, and HDL-C, followed by calculation of LDL-C using Friedewald’s formula.^166^ Several risk assessment systems have been developed, among which the widely applied Systemic Coronary Risk Estimation (SCORE),^167^ which is recommended by the European Guidelines on CVD prevention in clinical practice,^168^ because it is based on large European cohort datasets.

Quantities of total cholesterol, TG, HDL-C and LDL-C allow us to calculate (1) non-HDL-C (=total cholesterol – HDL-C) which quantity includes the cholesterol of all atherogenic lipoproteins (VLDL, IDL, LDL, CM, CM-remnants and Lp(a)) and (2) remnant-C (=total cholesterol – HDL-C – LDL-C) which quantity includes the cholesterol of all lipoproteins except LDL and HDL. Non-HDL-C is a global measure of all atherogenic lipoproteins and has been suggested as a predictor of CHD risk. LDL-C, a major component of non-HDL-C, is the current target to be treated and this is achieved with statins, ezetimibe and PCSK9 inhibitors. Remnant-C is included in non-HDL-C but has merit in being measured separately: these remnants, mostly TG-rich remnant lipoproteins (in the non-fasting state CM-remnants and IDL) contribute also to risk of CHD.^46^

Several studies confirm that non-HDL-C is superior to LDL-C in predicting CHD.^151,169^ In a prospective study participants with high non-HDL-C concentrations were at increased CHD risk, independent of LDL-C concentrations.^170^ An analysis of the treating to new targets (TNT) and Incremental Decrease in End Points Through Aggressive Lipid Lowering (IDEAL) trials found that on-treatment, concentrations of non-HDL-C and apoB100 were better predictors of CVD risk than LDL-C.^171^ Puri et al. reported that the achieved concentrations of on-treatment non-HDL-C and TG concentrations were closely associated with coronary atheroma progression and regression, irrespective of achieved concentrations of LDL-C, CRP and DM status.^172^

While the current clinical care pathway does indeed improve cardiovascular risk, considerable residual risk remains, particularly in women and individuals with remnant lipoprotein particles.

### Proposed cardiovascular precision care pathway

**Figure 6.**
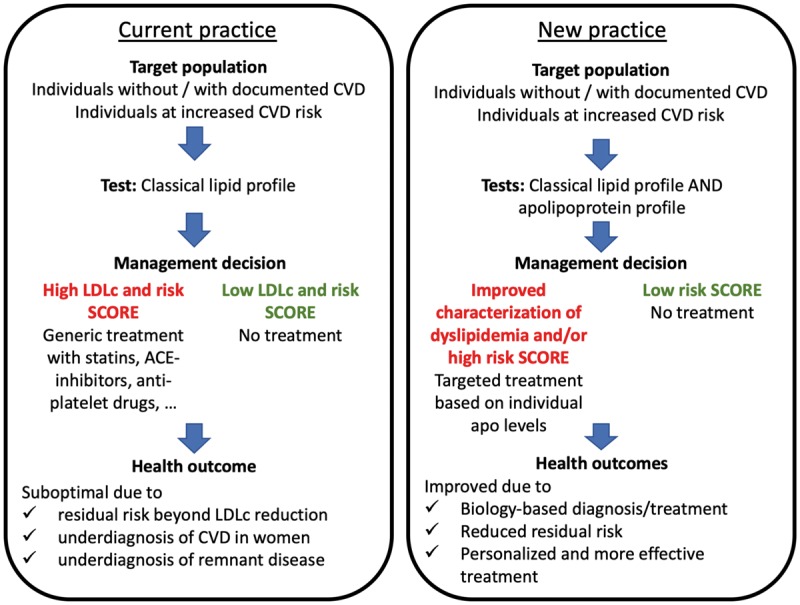
Clinical test-treatment pathways for CVD reduction according to current and new practices.*Source:* reproduced with permission from Elsevier.^7^

The use of novel markers to improve cardiovascular risk stratification as well as newly developed therapeutics targeting apoCIII and apo(a), pose a challenge for laboratory professionals. The use of novel markers based on improved understanding of pathophysiology would also help target therapy. Based on our current understanding of lipoprotein pathophysiology and the knowledge acquired from MRT-studies and protein-based outcome studies, we suggest that a multiplexed panel including apoA-I, apoB, apoCI, apoCII, apoCIII, apoE and apo(a) might allow improved risk stratification (see Figure 6 for a proposed clinical care pathway). Currently, a Consensus Conference Report from the American Diabetes Association and the American College of Cardiology suggested that measurement of apoB100 be added to measures of LDL-C and non-HDL-C in patients at high cardiometabolic risk, with target apoB100 concentrations set at <900 mg/L in high risk patients and <800 mg/L in highest risk patients.^154^ Moreover, two studies have reported that quantitation of Lp(a) in intermediate risk patients could potentially reclassify 15 to 40% of individuals into a lower or higher risk groups.^134,173^ It is highly likely that similar results will be obtained using apo(a). Guidelines from the European Atherosclerosis Society^1^ and the Canadian Cardiovascular Society^38^ recommend screening for apo(a) in patients with a family history of CVD in addition to quantitation of apoB100. While these guidelines originating from 2016 do not mention apoCs, we believe the very recent compelling evidence for the role of apoCs in CVD both at the genetic and protein concentration^106,174^ (sections on Genetic and Proteomic evidence for the role of apolipoproteins in CVD) warrants their inclusion in risk stratification.

Another group of patients that might benefit from a multiplexed apo panel in conjunction with conventional lipid measurements are individuals with a family history of CVD. For instance, individuals with FH typically suffer their first CVD event prior to the age of 50.^175^ Given that apo concentrations are mainly genetically defined (Figure 6) there may be a role for apo measurement in screening family members of young CVD patients as a first line strategy prior to genotyping. Therefore, it is likely that measurement of an apo panel in a single serum sample might be sufficient to characterize a particular familial dyslipidaemia. Indeed, recently the intra-individual variability of serum apo(a) concentrations was shown to be low.^176^

While there is substantial evidence that apos may be better predictors for CVD, there is as yet insufficient evidence from prospective outcome studies on the use of multiplexed apo panel as compared with a conventional lipoprotein panel. Therefore, we would currently propose the use of an apo multiplex panel as an add-on test, even though this will carry an associated additional health care cost. Future studies should be directed towards a comparison of the traditional lipoprotein panel and a multiplexed apo panel, so that ultimately, the apo panel could potentially replace the traditional lipoprotein panel. Based on the evidence outlined in the previous three sections, we believe a biology-driven multiplexed apo panel including apoA-I, apoB100, apoCI, apoCII, apoCIII, apoE and apo(a) will provide better CVD risk stratification and allow for targeted and more effective treatment (e.g., high apoCIII could be treated directly with anti-apoCIII ASO valonesorsen) than current cholesterol, TG, HDL-C and LDL-C measurement (Figure 6).

Inclusion of such a multiplexed apo panel in the clinical care pathway would be feasible from a technological perspective, as we previously developed an MS-based test that allows for the simultaneous quantitation of apos,^31^ and were recently able to show that this test can be delivered with stable analytical performance over a long period of time.^36^ The volume of serum required for the test is only 25 *µ*L. Although the current turnaround time of 48 hours to perform the multiplexed apo panel is considerably slower than that of the traditional lipid panel, this must be offset against the possibility of more accurate risk assessment.

## Conclusions and future outlook

The delivery of healthcare to the highest standard without a further increase in cost, requires that clinical practice should become more efficient. The current clinical care pathway for cardiovascular risk assessment is unsatisfactory, in that there remains considerable unexplained residual risk, even if LDL-C targets are met. It is time for a paradigm shift from traditional reactive medicine, often based on statin therapy, to proactive ‘P4 medicine’ in which disease mechanisms and systems biology allow for a targeted and patient-centric approach.^5,6^ We believe that the use of a multiplexed apo panel will allow better characterization of a patient’s dyslipidaemic status than that given by the traditional lipid profile (total cholesterol, TG, HDL-C, and calculated LDL-C). Apo profiling can be performed using an MS-based strategy, since the requirements for applying clinical proteomics, including apo profiling, in medical laboratories can be met using low resolution MS with applications that fulfil desirable performance criteria and that are standardized according to the calibration hierarchies outlined in ISO 17511. Because the apo profiles provide insight in the pathophysiology of the dyslipidaemia, it will facilitate the refining of therapeutic targets and will allow the use of more targeted therapies such as ASOs which may help reduce residual cardiovascular risk. In the near future it should be possible to demonstrate that apo profiling should be clinically effective and cost effective, and can be run with adequate throughput.

Further developments in risk stratification and tailoring of therapy may emerge in the near future. It is now clear that one gene can present itself in multiple proteoforms due to genetic modifications, alternative splicing and post-translational modifications.^177^ Because different proteoforms are likely to have different functionality, quantitation of proteoforms might be of importance, specifically in light of precision medicine and targeted therapeutics.^178^ Proteoforms have been found for several apos. Specifically, differential O-glycosylation of apoCIII was initially observed using isoelectric focusing,^179^ and, more recently, using intact protein MS analysis.^180^ Concentrations of sialylation on apoCIII are associated with lipid profiles in DM2,^181^ and lower concentrations of non- and mono-sialylated apoCIII were associated with larger LDL particles after dietary interventions.^182^ Proteoforms of apos CI and CII have also been reported,^183^ and an in-depth analysis of apoA-1 recently revealed large proteoform heterogeneity.^184^ Currently the clinical relevance of the proteoforms of apos for CVD are unknown, and more research is warranted. MS is an excellent technique for the in-depth characterization of an individual’s proteoform-profile,^178,185^ as mass-based differences either at the intact protein or at the peptide concentration can easily be identified. Specifically the introduction of high resolution MS-instruments, which can provide accurate mass information at resolutions >60,000, would be beneficial for the analysis of proteoforms.^186^ We believe that in the future, proteoform specific analysis in an extended apo-form panel might provide additional benefits, but such analyses are far from clinical implementation and require further technical development before clinical evaluation could be considered.

Overall, we believe that implementation of a multiplex apo panel for refined diagnosis of CVD would fit the unmet clinical need for enhancing stratified cardiovascular medicine. There is compelling evidence as outlined in this review, that the use of a biology-driven apo panel fits the concept of precision medicine, with potential benefit for patients and society. A carefully selected and biology-derived apo panel will likely enable tailored treatment of individual patients rather than standard treatment of average patients.
